# Molecular dynamics simulation analysis of the beta amyloid peptide with docked inhibitors

**DOI:** 10.6026/97320630018622

**Published:** 2022-07-31

**Authors:** Bandar Aloufi, Ahmad Mohajja Alshammari, Nawaf Alshammari, Mohammad Jahoor Alam

**Affiliations:** 1 Department of Biology, College of Science, University of Hail, Kingdom of Saudi Arabia

**Keywords:** Beta amyloid, Alzheimer, natural compounds, Docking, MD simulation

## Abstract

Beta amyloid peptide is widely studied due to its association with Alzheimer disease (AD). Various study reported that the accumulation of beta amyloid in brain cells leads to Alzheimer disease. Hence, Beta amyloid peptide could be a potential target of anti-AD therapy. Hence, it is of interest to develop potent inhibitors for Beta amyloid peptide in the context of Alzheimer disease (AD). We report the binding features of Ascorbic acid, Cysteine, Dithioerythriol, Dithiothreitol, Malic acid and α-Tocopherol with beta amyloid having binding energy values of -6.7, -6.5, -6.0, -6.5, -6.7 and - 7.0 kcal/mol respectively. The molecular docking of top-scoring compounds with beta amyloid suggests that amino acids such as ASP23, GLU22, Phe19, are crucial in binding. Molecular dynamics simulation study showed steady-state interaction of compounds with beta amyloid for further consideration.

## Background:

Alzheimer's disease (AD) is reported as neurodegenerative disease. It occurs due to accumulation of amyloid-beta peptide's in the brain which leads to neurotic plaques and neurofibrillary [1, 2]. Moreover, the presence of amyloid plaques leads to a massive loss of neurons in the brain so that patients suffered from memory loss and change of personality [3- 5]. Recently, it is reported that there are around 50 million population are suffering from AD, worldwide. It is also predicted that it will increase to reach 152 million by 2050. Many researches are still in progress to find out suitable treatment [6]. There are various inhibitors have been reported for beta amyloid [7, 8, 9]. Moreover, several diet components such as Ascorbic acid, Cysteine, Dithioerythriol, Dithiothreitol, Malic acid and α-tocopherol are reported to be playing an active role in suppression of risk of AD. Several studies have been performed so far to find out impact of food component on suppression of risk of AD [10]. However, molecular interaction between diet components and Amyloid beta peptide is still few reported. In the present work we studied that how these diet components (Ascorbic acid, Cysteine, Dithioerythriol, Dithiothreitol, Malic acid and α-tocopherol) interact with Amyloid beta peptide. We performed molecular docking study to find out structural interaction and best pose. Further, best pose was selected for molecular dynamics simulation study [11-16].

## Material and Methods:

### Protein Structure Preparation: 

We retrieved Beta amyloid 3D structure (PDBID: 1IYT) from the protein databank. Further, Complex 3D structure is refined into in monomer form using Discovery Studio Version 2020 [17].

### Database Collection and Refinement:

Dietary compounds (Ascorbic acid- CID 54670067 , Cysteine - CID 6419722, Dithioerythriol - CID 439352, Dithiothreitol - CID 446094, Malic acid - CID 525 and α-tocopherol - CID 1742129) were retrieved from online available PUBCHEM database (https://pubchem.ncbi.nlm.nih.gov/) [18, 19]. Next, we minimized and prepared all ligands for screening purpose by using "ligand preparation" tool available in Discovery studio version 2020.

### Molecular Docking:

The molecular docking was performed through AutoDock Vina tools to determine the receptor-ligand interactions [20, 21]. For ligand binding site of beta amyloid, we fixed the parameters of grid box with X=52, Y=56, Z=80 (Center grid box: X = 2.384, Y = -1.009, Z = 3.269; Spacing = 0.347Angstrom) dimensions. Moreover, we used AutoDock Vina tool carry out all the docking procedure with the predetermined parameters as mentioned above. Further, we visualized the receptor-ligand interaction by Discovery studio 4.0 clients [22]. We determined hydrophobic interactions and hydrogen bonds between dietary compounds using LIGPLOT+ online software [22-23].

### Molecular Dynamics (MD) Simulations:

We performed molecular dynamics (MD) simulation of the peptide-ligand complexes using GROMACS 5.1.4 package [24, 25]. We applied GROMOS 96 force field upon amyloid beta peptide while the ligand topologies were generated by online PRODRG server [26]. The complexes were solvated using simple point charge (SPC) water molecules in a rectangular box where every protein-ligand complex was placed in the center at least 1.0 nm from the box edges. To make the simulation system electrically neutral, required number of Mg+ and Cl− ions was added while 0.15 mol/L was set as the salt concentrations in all the systems. Using the steepest descent method, all the solvated systems were subjected to energy minimization for 5000 steps. Further, the NVT (constant number of particles, volume, and temperature) series and the NPT (constant number of particles, pressure, and temperature) series were conducted at a 300 K temperature and 1 atm pressure for duration of 100 ps (picoseconds) in the MD simulation selecting V‐rescale and Parrinello‐Rahman as the thermostat and barostat respectively. Finally, the production runs of six peptide-ligand complexes were performed for duration of 100 ns (nanoseconds) at 300 K temperature. Further, we compared all six complexes by various parameters such as root mean square deviation, root mean square fluctuation, radius of gyration, hydrogen bonding interaction and solvent accessible surface area. The results were plotted using the XMgrace tools [27-29].

### ADMET Property Prediction: 

All the dietary compounds (Ascorbic acid, Cysteine, Dithioerythriol, Dithiothreitol, Malic acid and α-tocopherol) used to predict drug-likeness, toxicity, and pharmacokinetic properties by uploading smiles structure, retrieved from PUBCHEM database on the pkCSM and Swiss ADME tools [30-35].

## Results and Discussion:

### Virtual Screening and Molecular Docking:

Natural dietary compounds from the PUBCHEM database were docked against beta amyloid peptide. We selected best pose according to low docking energy score. The docking energy scores are listed for each complex in Table 1(see PDF) and their corresponding protein-ligand interactions are shown in Figure 2(see PDF). Alpha-tocopherol showed the highest docking energy of -7.0 kcal/mol with amyloid-beta peptide. It formed 9 conventional hydrogen bonds with various residues (Phe19, Glu22 and Asp23) of the amyloid beta peptide. Glu22 and Asp23 were common interacting residues in 3 of the 6 complexes while interactions with Val12 and Gln15 were found in 2 complexes. Alpha-tocopherol formed a highest number of 5 hydrogen bonding interactions with the peptide as shown in Figure 3(see PDF). Also, high docking scores suggest that Alpha-tocopherol-beta amyloid complex is more active against amyloid-beta [14, 36-38].

### MD Simulation:

We performed MD simulations of the best hit docked protein–ligand for all six complexes usingGromacs2020 on the Linux platform. MD simulation was run up to 100 ns for all six complexes to study the structural dynamics of the receptor–ligand complex with time. Further, the simulation results were analyzed using the trajectory files, generated from MD simulation to get RMSD, RMSF, protein–ligand interactions.

### RMSD:

The RMSD (Root Mean Square Deviation) value defines mean deviation of the complexes with respect to time. Moreover, the average changes in an atom's displacement in the molecular conformation can be observed by RMSD analysis. It observed from panel A, Figure 4(see PDF) that for all the six complex, RMSD was found within the range of 0.25Å and 1.5 Å. This suggests all compounds form a stable complex with beta-amyloid. Moreover, we monitored back bone atoms for structural fluctuations, compactness, protein-ligand interactions sites and stability. We observed that among six complexes, Amyloid beta-ascorbic acid complex had comparatively highest RMSD values (1.5 Å). Further, Amyloid beta- alpha-tocopherol had a comparatively lowest RMSD value. Thus, it indicates that Amyloid beta-alpha-tocopherol complex has more stability.

### RMSF:

The Root Mean Square Fluctuation (RMSF) defines local protein mobility in the protein–ligand complex. It determines flexibility of a protein region in protein ligand complex. The RMSF plot (Figure 4, panel B - see PDF) indicates that in case of Amyloid beta-alpha-tocopherol complex, there is comparatively minimal fluctuations in the protein structure. It suggests that ligand binding sites in beta-amyloid protein remained rigid throughout the simulation.

### Number of Hydrogen Bonds (H-Bond Number):

H-bonds indicate the robustness of the complex. The minimum cut-off value <2.5nm is used to find out H-bond in complexes. We found that number of hydrogen bonds in complexes of Amyloid beta and above dietary compounds are up to 5 which indicates more stable complex formation (Figure 4, panel C - see PDF).

### Radius of Gyration (Rg):

The Radius of Gyration (Rg) is used to characterize parameters which influence changes in protein structures. The Rg values of complexes between beta-amyloid and six ligands were not much vary significantly throughout simulation as shown in Figure 4, panel D(see PDF). The Rg value observed in between 1 and 1.9 nm, indicates ligands had little influence on protein structures. It also documented that Rg value of complex Amyloid beta-alpha-tocopherol was lower and little fluctuations throughout the 100ns of simulation. This indicates that Amyloid beta-alpha-tocopherol complex structures are more compact.

### Solvent Accessible Surface Area:

Solvent Accessible Surface Area defines the compactness of the complex. We observed that solvent accessible surface area is around 35 nm. This lower value of solvent accessible surface area suggests that all complexes between Amyloid beta and above dietary compounds are form stable complex (Figure 4, panel E - see PDF).

### Predicted Absorption, Distribution, Metabolism, Excretion and Toxicity (ADMET) properties:

The predicted value of ADMET properties (as shown Table: 2 - see PDF) have been calculated using online tools. The predicted values indicate favorable drug-likeness properties of these dietary compounds.

## Conclusions:

We document the molecular binding and simulation features of Ascorbic acid, Cysteine, Dithioerythriol, Dithiothreitol, Malic acid and α-tocopherol with beta amyloid for further consideration in the context of AD.
